# Morphological identification keys for adults of sand flies (Diptera: Psychodidae) in Sri Lanka

**DOI:** 10.1186/s13071-020-04305-w

**Published:** 2020-09-07

**Authors:** Tharaka Wijerathna, Nayana Gunathilaka

**Affiliations:** Department of Parasitology, Faculty of Medicine, University of Kelaniya, Ragama, Sri Lanka

**Keywords:** Sand flies, Taxonomy, Morphology, Identification, Vectors

## Abstract

**Background:**

Phlebotomine sand flies are a medically important group of insects that is responsible for the transmission of leishmaniasis. Surveillance plays a major role in vector control programmes through exploring species abundance, potential entomological risk and designing appropriate control measures. In field surveillance programmes of such nature, morphological identification of vector species is of paramount importance. However, in Sri Lanka, there is no published taxonomic key available for the identification of leishmaniasis vectors.

**Method:**

Identification keys for both male and females of the sand flies recorded in Sri Lanka were developed using morphological features. Main identification features were compared with the original observation of specimens collected from surveys and the use of published literature. Photographic illustrations of morphological features are included with the intention of making the keys user-friendly for non-taxonomists.

**Results:**

A total of 22 sand fly species (Diptera: Psychodidae) of the genera *Phlebotomus* and *Sergentomyia* reported in Sri Lanka from 1910 to 2019 are included in the present work.

**Conclusion:**

This simplified key, along with photographs taken from specimens would be beneficial to the health staff, entomologists and research staff who deal with leishmaniasis control programmes and vector-related studies.
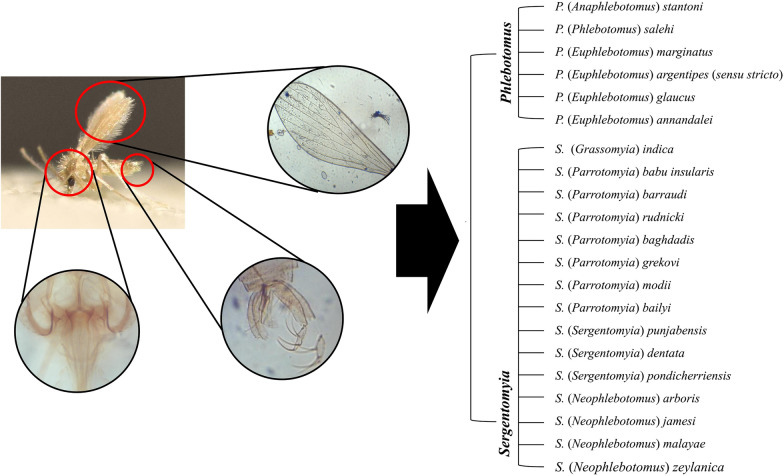

## Background

Leishmaniasis is a complex disease with a range of clinical and epidemiological features. This disease is caused by protozoan parasites of the genus *Leishmania* that can infect numerous mammals, including humans [1]. These parasites are mainly transmitted by phlebotomine sand flies (Diptera: Psychodidae) [2, 3]. Currently, 98 countries are endemic for at least one form of leishmaniasis with more than 58,000 visceral leishmaniasis and 220,000 cutaneous leishmaniasis patients per year [4]. The World Health Organization (WHO) has set up a target to eliminate visceral leishmaniasis, from the Indian subcontinent by the year 2020 [5].

Around 927–1000 species of sand flies have been reported in tropical, temperate and desert climatic conditions all over the world [6–8]. These species belong to three New World genera (*Brumptomyia*, *Lutzomyia* and *Warileya*) and three old world genera (*Phlebotomus*, *Chinius* and *Sergentomyia*) [9]. In other countries, the prevalence and abundance of sand flies have been assessed and the checklists of species are updated frequently. However, in Sri Lanka, information on sand flies was limited until the recent past. The first report of sand flies from Sri Lanka was by Annandale in 1910, who recorded the presence of *Phlebotomus argentipes* and *Sergentomyia zeylanica* [10]. Later studies reported the presence of *Phlebotomus stantoni*, *Sergentomyia arboris* [11] and *Sergentomyia punjabensis* [12].

The first local case of leishmaniasis from Sri Lanka was reported in 1992 [13]. It took only about 15 years to become a significant health problem in the country. In 2008, the Ministry of Health of Sri Lanka declared leishmaniasis as a notifiable disease in the country. Later in the period from 2009 to 2016, nearly 8487 cases were notified representing at least one case from all 25 districts of the country. Some districts had an increasing trend of the disease prevalence with more than 100 new cases each year [14, 15]. Therefore, more attention was drawn toward diagnostic, treatment, and vector-related studies of leishmaniasis by the scientific community. Regarding the vector species, a total of 12 species have been recorded from the country, i.e. *Sergentomyia jamesi*, *Sergentomyi indica*, *Sergentomyia barraudi*, [16], *Phlebotomus salehi* [17], *Sergentomyia dreyfussi*, *Sergentomyia malayae*, *Sergentomyia baghdadis*, *Sergentomyia bailyi*, *Sergentomyia grekovi*, *Sergentomyia modii*, *Sergentomyia rudnicki* and *Sergentomyia dentata* [18]. The recently published checklist for Sri Lankan sand flies has indicated the presence of 20 distinct species in the country [19].

At present leishmaniasis is notified even from the wet zone areas of the country, which were previously regarded as non-endemic areas. Therefore, this warrants a proper control programmes and efforts. However, at the regional level, there are no proper surveillance data available on the vector distribution and abundance to design vector control strategies. Lack of knowledge and trained staff for field surveys/vector identification are the main limitations. Traditional field taxonomy based on morphological characteristics remains the backbone of all vector control programmes. However, local public health workers and scientists concerned with the study and/or control of the vectors in Sri Lanka have no morphological keys to Sri Lankan sand fly species. This has become a major challenge in the control efforts as the accurate identification of vectors is crucial for vector incrimination.

Therefore, this morphological identification key has been prepared to minimize the above limitations in the sand fly taxonomic studies. The key was prepared for adults of the recorded sand fly species in Sri Lanka as simply as possible, using the most important characters. Hopefully, this will enable public health workers, students and entomologists for rapid and accurate identification of sand flies to the genus and species levels.

## Methods

### Collection of samples

Samples were collected using Cattle Baited Trap, light trap and sticky trap collections in the selected areas in Kurunegala District, Sri Lanka. Further, samples obtained from Anuradhapura, Mannar, Trincomalee, Jaffna, Hambanthota, and Gampaha Districts through previous research studies and entomological teams at the district level were also assessed (Additional file 1: Table S1).

### Storing and preservation of field-caught samples

Live sand flies were sacrificed using a killing jar with 70% of chloroform and preserved in 25 ml falcon tubes containing 70% v/v ethanol solution [6]. The sand flies trapped in sticky traps were carefully removed using a fine paintbrush wetted with acetone and preserved in 70% ethanol.

### Dehydration

The specimens were dehydrated by placing them in each of the 70% ethanol, 90% ethanol, absolute ethanol, and xylene for 5 min. Specimens were cleared in 10% lactophenol for 1–2 hours.

### Identification of sand flies

Phlebotomine sand fly specimens were examined under a dissecting microscope (Lebomed CZM4 Binocular Zoom Microscope, Lobo American Inc, USA) at 10–40× and separated according to sex. Specimens were dissected on a glass slide separating the terminal part of the abdomen, wings and entire head with a fine needle and mounted in Berleseʼs medium for later identification. The sand flies were identified based on morphometric and meristic characters [6, 20]. The morphometric measurements were taken using an ocular micrometer attached to a binocular microscope.

### Morphological characterization

The morphological characteristics used here were based on original observations and previous usage in the literature [6, 7]. The publications of Lewis [6], Kalra & Bang [7], Wijerathna & Gunathilaka [20] and Ilango [21] were consulted during the construction of the keys. Morphological characteristics were cross-checked through published literature and examining the reference specimens archived at the Department of Parasitology, Faculty of Medicine, University of Kelaniya, Sri Lanka.

The following basic features were considered in the morphological characterization: *Head* (length and width; longitudinal diameter of the eye and distance between the eye on dorsal side of the head; number and distribution of the hair sockets on the dorsal side of the head; length of the labrum; shape of the labral tip; shape of the maxilla and the number of lateral and ventral teeth; shape of the mandible; presence and number of the serration on hypopharynx; length of antennal segment 3 (A3), relation A3/A3+4, relation A3/labrum (A3/L), antennal formula; length of the ascoid on A4 and relation ascoid 4/A4, and papilla formula); *Palps* (palpal formula; relative lengths of palpal segments, which can be expressed in relation to first segment; total length of the palp; position of the Newstead’s scales); *Cibarium* (presence and number of horizontal and vertical teeth, their position and arrangement; presence, shape, and color of the pigment patch; presence of the chitinous arch, shape of the cibarium and its ventral plate; presence and number of dorsal bulges). *Pharynx* (shape of the pharynx relation of its length to width; arrangement and development of pharyngeal armature elements); *Thorax* (length of the mesonotum; pigmentation of different sclerites; presence or absence of the pleural hairs); *Legs* (length of the hind leg, relation of hind leg to wing length; length of the femur, tibia and every tarsal segment, lengths of femur and hind tarsus; presence of short spines on the femora); *Wing* (length and width; length of R2 (from the fork to the apex); length of R2+3; relation R2/R2+3 (wing index); length of R2+3+4; length of R1 (from the fork R2+3 to the apex of R1); distance between the forms R2+3+4 and M1+2); *Abdomen* (presence or absence of erect or recumbent hairs on abdominal tergites 2–6; pigmentation of the abdominal tergites; shape of the spermathecal ducts; shape of the postgenital plate; shape of the furca).

Generally, two or more primary characteristics, identified through this characterization are used in each step of the key, to make them user-friendly for field taxonomists. The taxonomic grouping under genera, subgenera, and at the species level are in accordance with the International Code for Zoological Nomenclature [22].

## Results

**Fig. 1:**
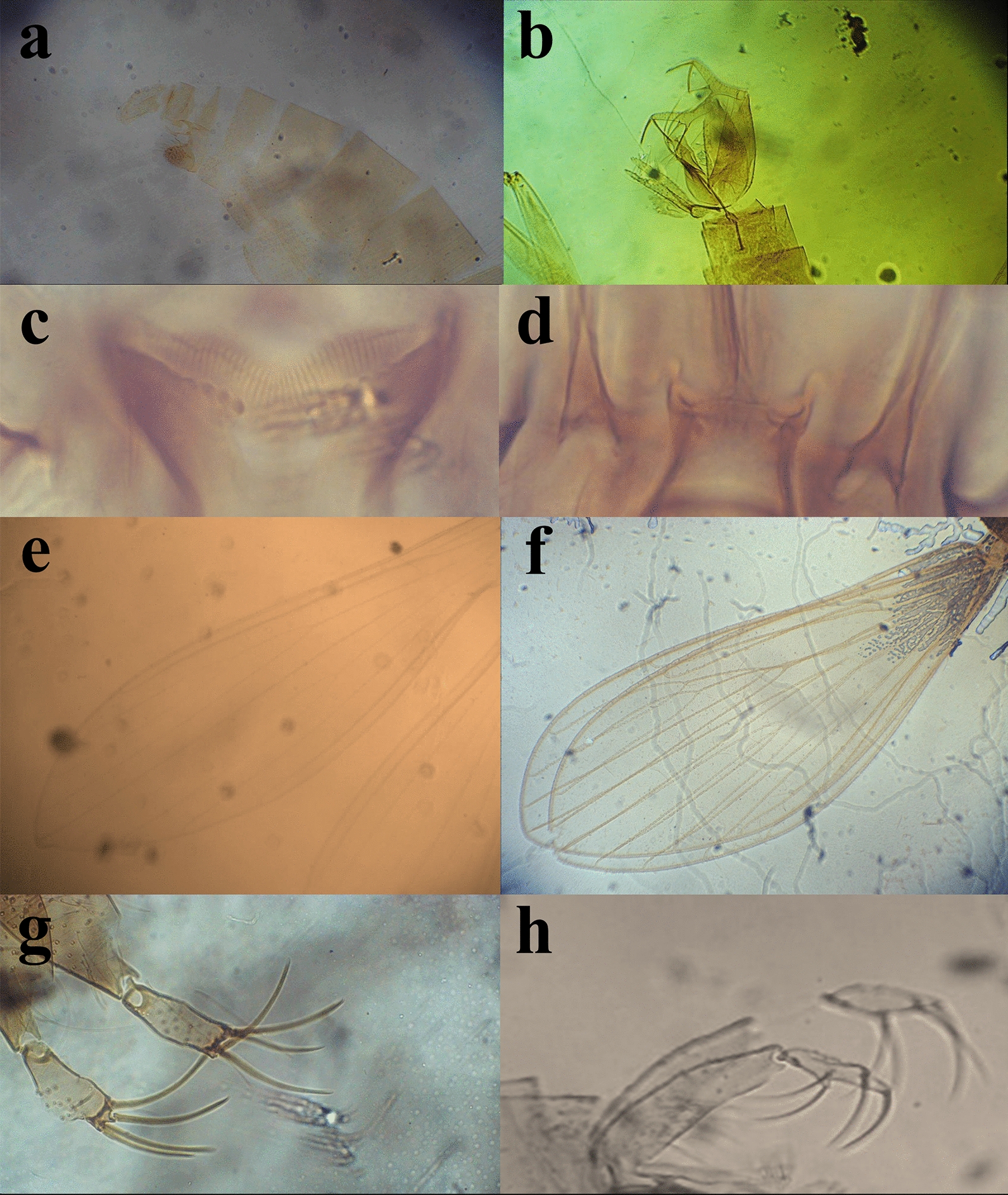
Morphological features used for sex and genus level discrimination of sand flies. **a** Terminalia of a female sand fly. **b** Terminalia of a male sand fly. **c** Cibarium of *Sergentomyia babu insularis*. **d** Cibarium of *Phlebotomus argentipes*. **e** Wing of *Sergentomyia zeylanica.* **f** Wing of *Phlebotomus argentipes*. **g** Gonostyle of *Sergentomyia punjabensis*. **h** Gonostyle of *Phlebotomus stantoni*

**Fig. 2:**
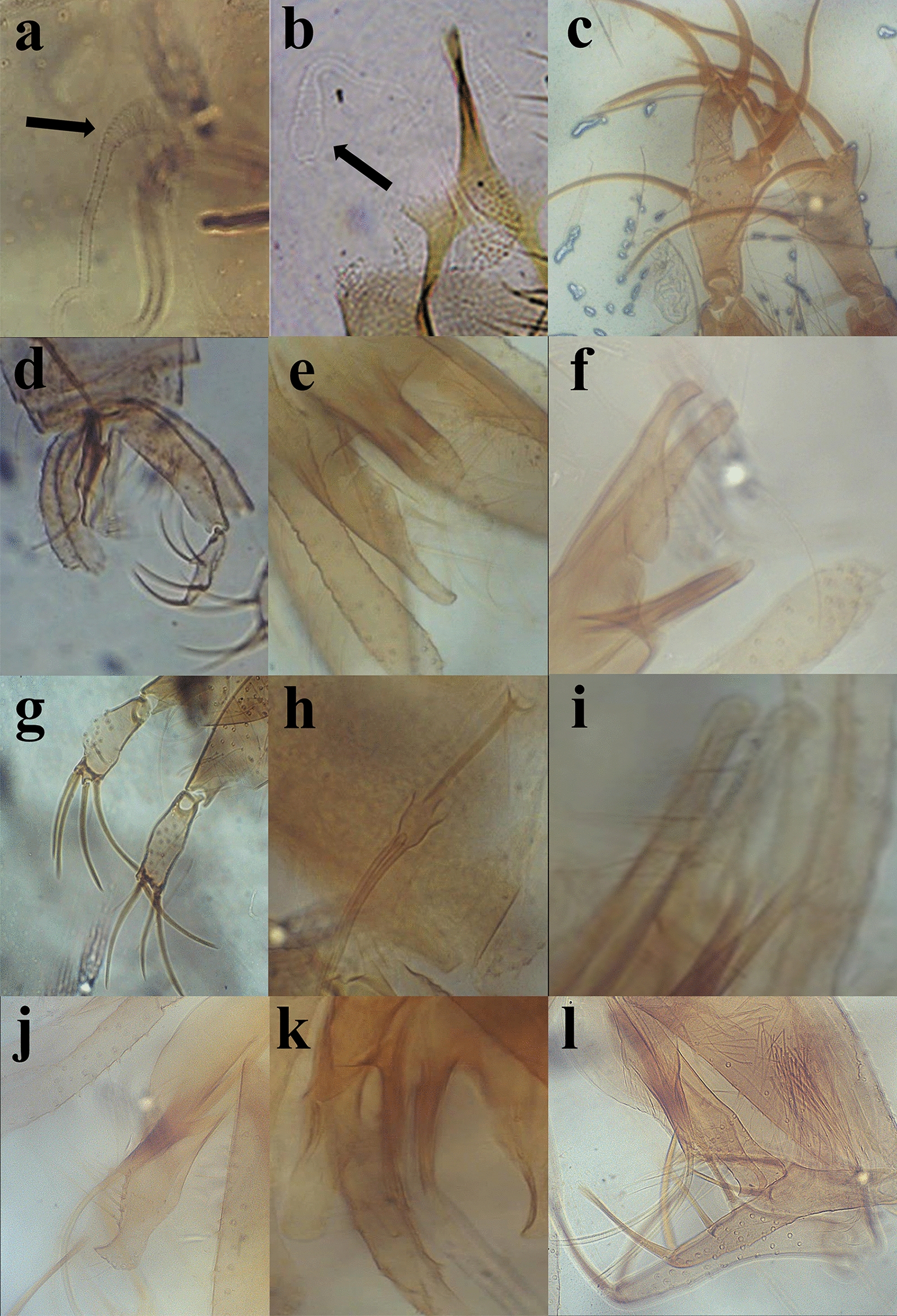
Terminal structures of sand flies. **a** Spermatheca of *Phlebotomus stantoni*. **b** Spermatheca of *Phlebotomus argentipes.* **c** Gonostyle of *P. argentipes.* **d** Gonostyle of *P. stantoni.* **e** Aedeagus of *Sergentomyia punjabensis.* **f** Paramere and aedeagus of *Sergentomyia babu insularis.* **g** Gonostyle of *S. punjabensis.* **h** Genital filaments of *Sergentomyia arboris.* **i** Paramere of *S. arboris.* **j** Paramere and aedeagus of *Sergentomyia zeylanica.* **k** Aedeagus of *S. arboris.* **l** Coxite and gonostyle of *S. zeylanica*

**Fig. 3:**
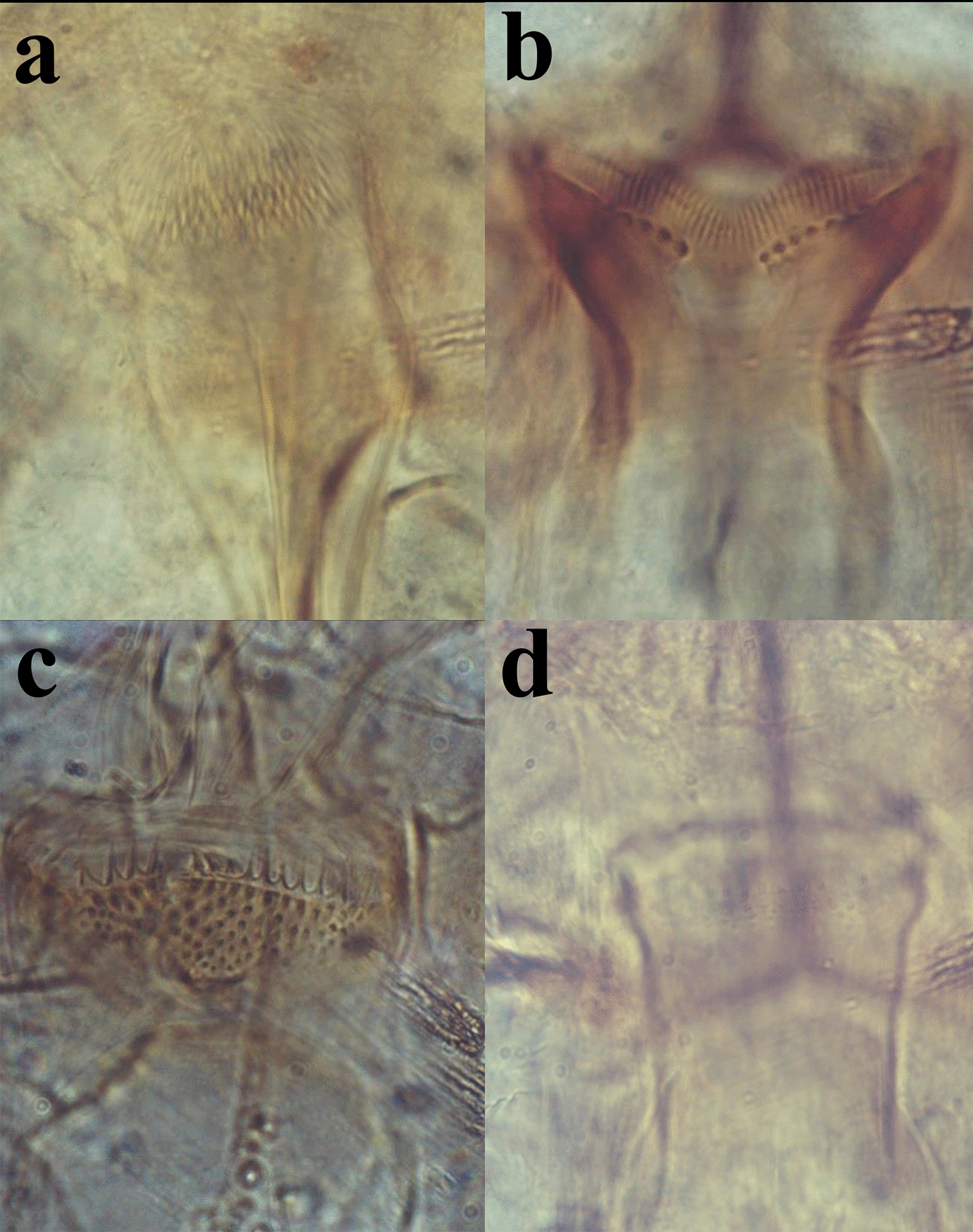
Cephalic structures of sand flies. **a** Pharynx of *Sergentomyia babu insularis.* **b.** Cibarium of *S. babu insularis* **c** Cibarium of *Sergentomyia arboris.* **d** Cibarium of *Sergentomyia zeylanica*

**Fig. 4:**
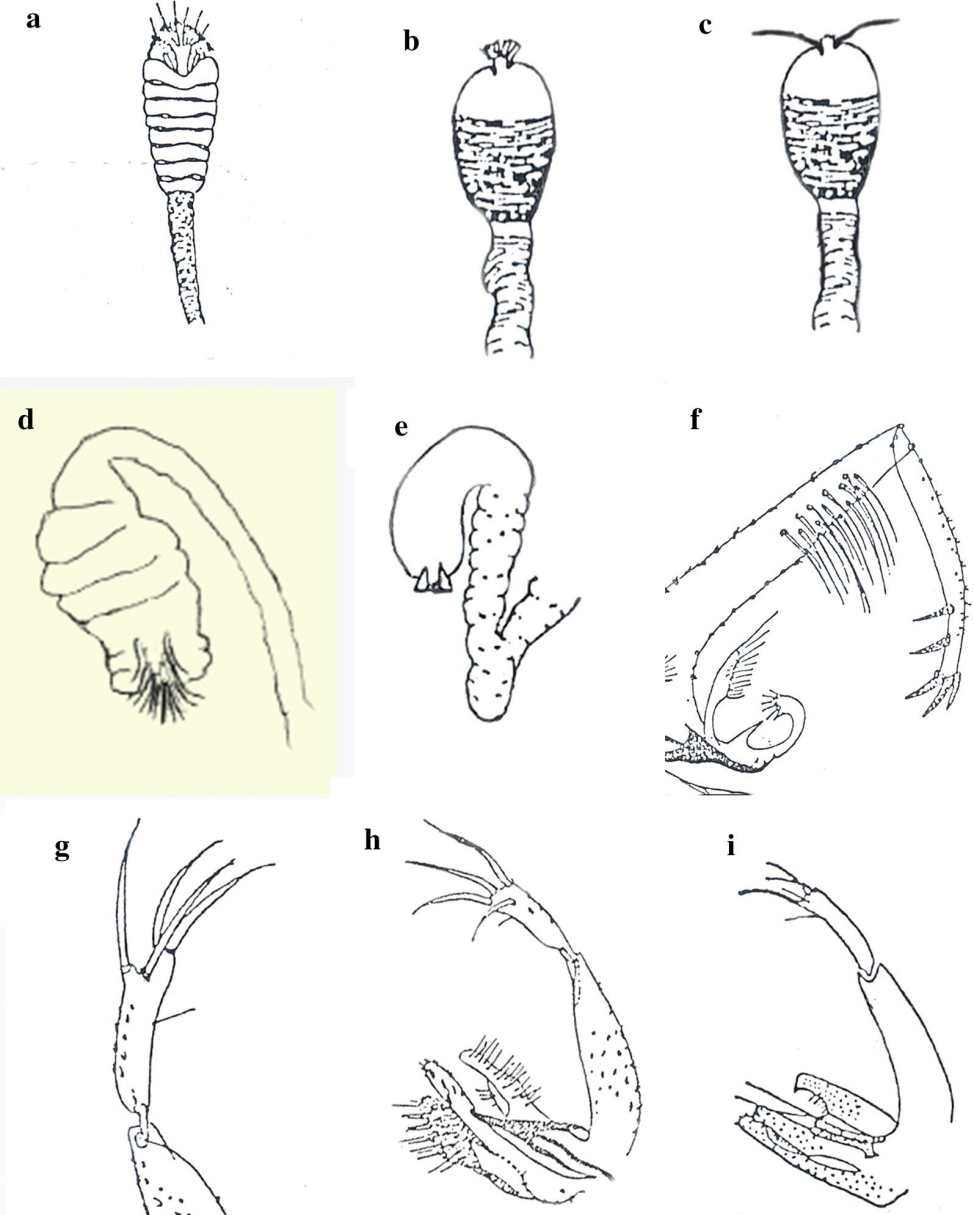
Illustrations of sand fly terminal structures adapted from previous publications [6, 7, 21]. **a** Spermatheca of *Phlebotomus salehi.* **b** Spermatheca of *Sergentomyia indica*. **c** Spermatheca of *Sergentomyia dreyfussi.* **d** Spermatheca of *Sergentomyia malayae*. **e** Spermatheca of *Sergentomyia punjabensis.* **f** Gonostyle of *P. salehi*. **g** Gonostyle of *Sergentomyia dentata.* **h** Paramere of *S. dreyfussi*. **i** Paramere of *S. indica*

**Fig. 5:**
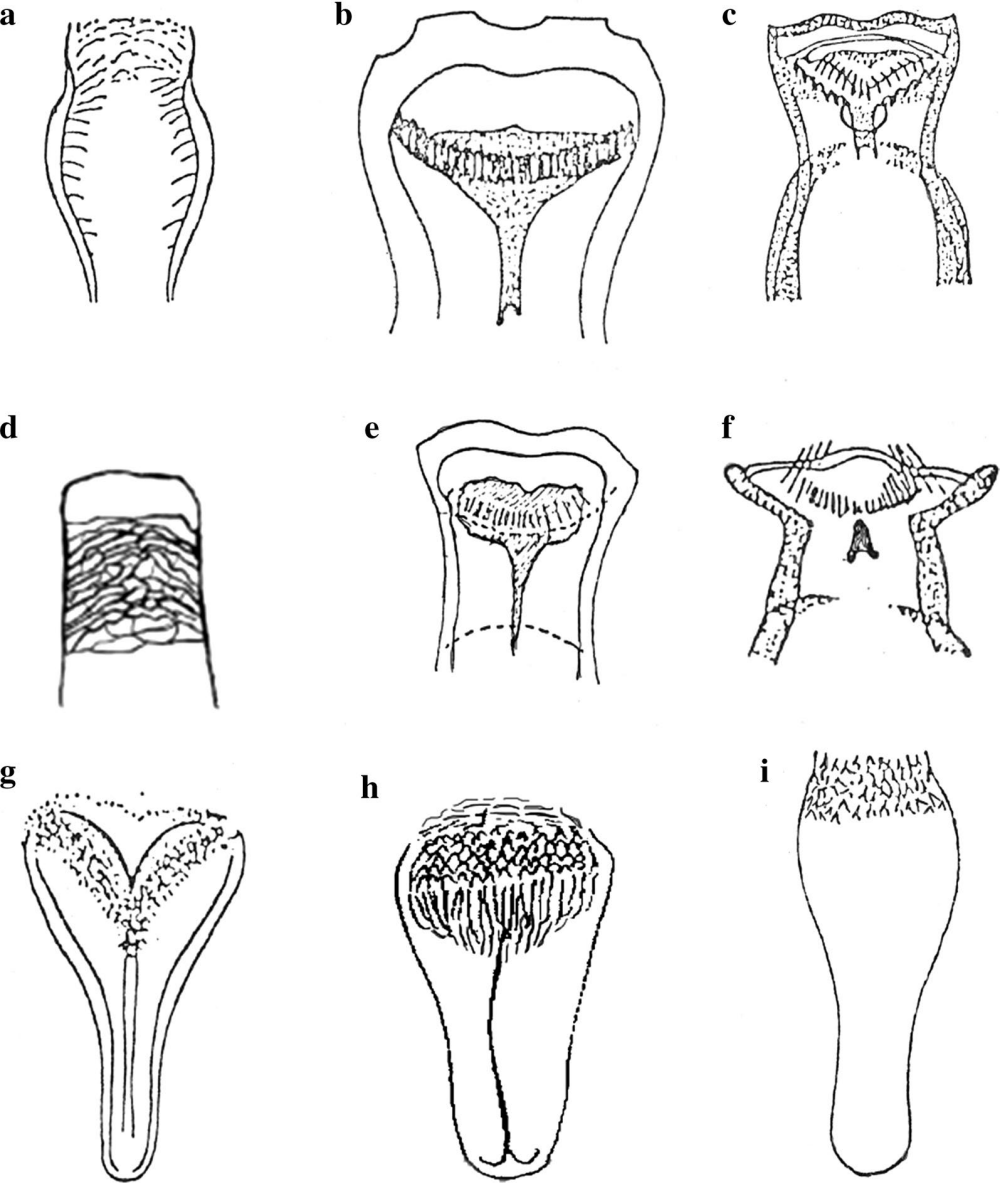
Illustrations of sand fly cephalic structures adapted from previous publications [6, 7, 21]. **a** Pharynx of *Sergentomyia baghdadis.* **b** Cibarium of *Sergentomyia barraudi.* **c** Cibarium of *S. baghdadis.* **d** Pharynx of *Sergentomyia grekovi.* **e** Cibarium of *Sergentomyia modii.* **f** Cibarium of *Sergentomyia bailyi.* **g** Pharynx of *Sergentomyia dentata.* **h** Pharynx of *Sergentomyia pondicherriensis.* **i** Cibarium of *Sergentomyia jamesi*

The present work illustrates the identification keys to adults of 22 sand fly species (Diptera: Psychodidae) which have been recorded in Sri Lanka. Females are characterized by terminal end lacking clasping structures and males are characterised by terminal end with clasping structures. Identification keys for female and male sand flies are provided in Tables 1 and 2, respectively. The main morphological features of taxonomic importance are illustrated in Figs. 1, 2, 3, 4 and 5).

**Table 1:** Key to sand fly species in Sri Lanka based on female morphology

Wings broader, asymmetrical along the length (Fig. 1f). Cibarium unarmed or with scattered spicules without a pigment patch (Fig. 1d)		Genus *Phlebotomus* Loew, 1845
Wings narrow, lanceolate, symmetrical along the length (Fig. 1e). Cibarium with one or more rows of teeth, pigment patch usually present (Fig. 1c)		Genus *Sergentomyia* (Franca & Parrot, 1920)
Genus *Phlebotomus*		
1a	Head of spermatheca with a distinct neck; spermatheca spindle-shaped (Fig. 2a)	*P.* (*Anaphlebotomus*) *stantoni* (Newstead, 1914)
1b	Head of spermatheca without a distinct neck (Figs. 2b, 4a); spermatheca not spindle shaped	2a
2a	Spermatheca with 7–8 segments; apical segment not enlarged (Fig. 4a)	*P.* (*Phlebotomus*) *salehi* Mesghali, 1965
2b	Spermatheca with 15–17 segments; apical segment enlarged (Fig. 2b)	3a
3a	Thorax dorsum brown, sides dark	*P.* (*Euphlebotomus*) *marginatus* Annandale, 1910
3b	Thorax dorsum black, sides pale	4a [*P.* (*Euphlebotomus*) *argentipes* Annandale & Brunetti, in Annandale 1908 (*sensu lato*)]
4a	Wing overlap (R_1_ overlap with R_2_/ complete length of R_2_) < 0.2; wing index (R_2_/R2_+3_) < 0.2; ascoid:antennal flagellomere ratio > 0.5	*P.* (*Euphlebotomus*) *glaucus* Mitra & Roy, 1953
4b	Wing overlap ≥ 0.2; wing index > 0.2; ascoid:antennal flagellomere ratio < 0.5	5a
5a	Wing overlap = 0.2; wing index = 2.0; ascoid:antennal flagellomere ratio > 0.4	*P.* (*Euphlebotomus*) *argentipes* Annandale & Brunetti, 1908 (*sensu stricto*)
5b	Wing overlap > 0.2; wing index < 2.0; ascoid:antennal flagellomere ratio < 0.4	*P.* (*Euphlebotomus*) *annandalei* Sinton, 1923
Genus *Sergentomyia*		
1a	Spermatheca with capsule (Fig. 4b, c), not tubular	2a
1b	Spermatheca without capsule, tubular (Fig. 4d)	10a
2a	Capsule of spermatheca with numerous spicules or striations (Fig. 4b, c)	3a [subgenus *Grassomyia*]
2b	Capsule of spermatheca smooth	4a [subgenus *Parrotomyia*]
3a	Tip of spermatheca with minute projections (Fig. 4b)	*S.* (*Grassomyia*) *indica* (Theodor, 1931)
3b	Tip of spermatheca with small projections (Fig. 4c)	*S.* (*Grassomyia*) *dreyfussi* Parrot, 1933
4a	Pharynx with distinct pointed teeth (Fig. 3a)	5a
4b	Pharynx with fine spicules or none (Fig. 5a)	7a
5a	Cibarium with a deep notch in hind end of ventral plate (Fig. 3b)	*S.* (*Parrotomyia*) *babu insularis* (Theodor, 1938)
5b	Cibarium without a notch in hind end of ventral plate (Fig. 5b)	6a
6a	Pharynx broad with numerous finely pointed teeth; cibarium with 40–70 teeth; tip of pigment patch bifid, ragged or fernestrated (Fig. 5b)	*S.* (*Parrotomyia*) *barraudi* (Sinton, 1929)
6b	Without this combination	*S.* (*Parrotomyia*) *rudnicki* Lewis, 1978
7a	Cibarium with a deep notch (Fig. 5c)	*S.* (*Parrotomyia*) *baghdadis* (Adler & Theodor, 1929)
7b	Cibarium without a deep notch	8a
8a	Pharynx with well-defined scales (Fig. 5d)	*S.* (*Parrotomyia*) *grekovi* (Khodukin, 1929)
8b	Pharynx without scales	9a
9a	Cibarium with pigment patch and 17 hind teeth; pharynx with long teeth (Fig. 5e)	*S.* (*Parrotomyia*) *modii* (Lewis, 1978)
9b	Cibarial pigment patch is small or absent (Fig. 5f); pharynx with spiculate ridges	*S.* (*Parrotomyia*) *bailyi* (Sinton, 1931)
10a	Spermatheca smooth (Fig. 4e)	11a [subgenus *Sergentomyia*]
10b	Spermatheca with striations (Fig. 4d)	13a [subgenus *Neophlebotomus*]
11a	Pharynx broad at posterior end with a deep constriction at base (Fig. 5g)	*S.* (*Sergentomyia*) *punjabensis* (Sinton, 1927)
11b	Pharynx barrel-shaped at posterior end and lacking a deep constriction at the base (Fig. 5h, i)	12a
12a	Posterior margin of pharyngeal armature convex (Fig. 5h)	*S.* (*Sergentomyia*) *dentata* (Sinton, 1933)
12b	Posterior margin of pharyngeal armature straight (Fig. 5i)	*S.* (*Sergentomyia*) *pondicherriensis* Srinivasan & Jambulingam, 2010
13a	Cibarium with about 8 rows of fore teeth (Fig. 3c); labrum 0.13–0.15 times the length of wing	*S.* (*Neophlebotomus*) *arboris* (Sinton, 1931)
13b	Cibarium with one or more rows of teeth; labrum > 0.11 times the length of wing	14a
14a	Cibarium without row of teeth; labrum 0.11 times the length of wing	*S.* (*Neophlebotomus*) *jamesi* Lewis, 1978
14b	Cibarium with 3 rows of fore teeth (Fig. 3d)	15a
15a	Labrum 0.18–0.20 times length of wing; R_2_/R_2+3_ < 2.06	*S.* (*Neophlebotomus*) *malayae* (Lewis, 1957)
	Labrum 0.13–0.15 times length of wing; R_2_/R_2+3_ > 2.06	*S.* (*Neophlebotomus*) *zeylanica* (Annandale, 1910)

**Table 2:** Key to sand fly species in Sri Lanka based on male morphology

Style with 4 or 5 spines; not all spines terminal (Fig. 1b)		Genus *Phlebotomus* Loew, 1845
Style with 4–5 spines, usually terminal; if not all spines terminal, 2 spines terminal and 2 sub-terminal, often in pairs (Fig. 1g)		Genus *Sergentomyia* (Franca & Parrot, 1920)
Genus *Phlebotomus*		
1a	Paramere with 2 long dorsal processes; style long, with 5 short spines (Fig. 4f)	*P.* (*Phlebotomus*) *salehi* Mesghali, 1965
1b	Paramere without processes or with short ventral processes; style long or short, with long spines (Fig. 2c, d)	2a
2a	Parameres tri-lobed. Style with 4 spines (Fig. 2d)	*P.* (*Anaphlebotomus*) *stantoni* (Newstead, 1914)
2b	Parameres with 2 ventral processes; style with 5 spines, rarely 6 spines (Fig. 2c)	3a [*P.* (*Euphlebotomus*) *argentipes* Annandale & Brunetti, in Annandale, 1908 (*sensu lato*)]
3a	Gonocoxite: gonostyle ratio < 1.5	*P.* (*Euphlebotomus*) *glaucus* Mitra & Roy, 1953
3b	Gonocoxite: gonostyle ratio > 1.5	4a
4a	Gonocoxite: gonostyle ratio > 1.65	*P.* (*Euphlebotomus*) *argentipes* Annandale & Brunetti, 1908 (*sensu stricto*)
4b	Gonocoxite: gonostyle ratio > 1.75	(*Euphlebotomus*) *annandalei* Sinton, 1923
Genus *Sergentomyia*		
1a	Aedeagus thick, finger-shaped (Fig. 2e)	2a [subgenus *Sergentomyia*]
1b	Aedeagus gradually tapering to the end (Fig. 2f)	4a
2a	Style with 2 terminal and 2 subterminal spines (Fig. 4g); cibarial teeth not uniform in size	*S.* (*Sergentomyia*) *dentata* (Sinton, 1933)
2b	Style with 4 terminal spines and no subterminal spines (Fig. 2g); cibarial teeth uniform in size	3a
3a	Cibarial teeth arranged in 2 rows	*S.* (*Sergentomyia*) *pondicherriensis* Srinivasan & Jambulingam, 2010
3b	Cibarial teeth arranged in a single row	*S.* (*Sergentomyia*) *punjabensis* (Sinton, 1927)
4a	Genital filaments with dilated ends; A3 without ascoid	5a [subgenus *Grassomyia*]
4b	Genital filaments with narrow ends (Fig. 2h); A3 with one ascoid	6a
5a	Paramere with rounded end (Fig. 4h)	*S.* (*Grassomyia*) *indica* (Theodor, 1931)
5b	Paramere with hooked end (Fig. 4i)	*S.* (*Grassomyia) dreyfussi* Parrot, 1933
6a	Paramere with hairy ventral tubercles (Fig. 2i)	7a [subgenus *Neophlebotomus*]
6b	Paramere without ventral tubercles (Fig. 2j)	9a [subgenus *Parrotomyia*]
7a	Aedeagus length *c.*10 times mid-width of shaft (Fig. 2k)	*S.* (*Neophlebotomus*) *arboris* (Sinton, 1931)
7b	Aedeagus length *c.*5 times mid-width of shaft	8a
8a	Outer hairs of the coxite evenly spaced	*S.* (*Neophlebotomus*) *malayae* (Lewis, 1957)
8b	Outer hairs of the coxite not evenly spaced, some of the hairs concentrated (Fig. 2l)	*S.* (*Neophlebotomus*) *zeylanica* (Annandale, 1910)
9a	Style *c.*4 times as long as thick; all spines on style	*S.* (*Parrotomyia*) *barraudi* (Sinton, 1929)
9b	Style 5 or 6 times as long as thick; spines on style not always apical	10a
10a	Cibarial fore teeth well developed	*S.* (*Parrotomyia*) *grekovi* (Khodukin, 1929)
10b	Cibarial fore teeth not well developed	11a
11a	Antennal segment 3 > 0.25 mm in length	*S.* (*Parrotomyia*) *rudnicki* Lewis, 1978
11b	Antenna segment 3 < 0.20 mm in length	*S.* (*Parrotomyia*) *babu insularis* (Theodor, 1938)

Four species of the genus *Phlebotomus* are reported under three subgenera: *Anaphlebotomus*, *Euphlebotomus* and *Phlebotomus*. Sixteen species of the genus *Sergentomyia* are reported. These species are classified into four subgenera: *Grassomyia*, *Neophlebotomus*, *Parrotomyia* and *Sergentomyia.* The species included in the key are as follows:

Genus *Phlebotomus*: *Phlebotomus* (*Anaphlebotomus*) *stantoni* Newstead, 1917; *P.* (*Euphlebotomus*) *argentipes* Annandale & Brunetti in Annandale, 1908 (*sensu lato*); *P.* (*Phlebotomus*) *marginatus* Annandale, 1910; and *P.* (*Phlebotomus*) *salehi* Mesghali, 1965.

Genus *Sergentomyia*: *Sergentomyia* (*Grassomyia*) *dreyfussi turkestanica* Theodor & Mesghali, 1964; *S.* (*Grassomyia*) *indica* Theodor, 1931; *S.* (*Neophlebotomus*) *arboris* Sinton, 1931; *S.* (*Neophlebotomus*) *jamesi* Lewis, 1978; *S.* (*Neophlebotomus*) *zeylanica* Annandale, 1910; *S.* (*Neophlebotomus*) *malayae* Lewis, 1957; *S.* (*Parrotomyia*) *babu* var. *insularis* Theodor, 1938; *S.* (*Parrotomyia*) *baghdadis* Adler & Theodor, 1929; *S.* (*Parrotomyia*) *bailyi* Sinton, 1931; *S.* (*Parrotomyia*) *baghdadis barraudi* Sinton, 1929; *S.* (*Parrotomyia*) *grekovi* Khodukin, 1929; *S.* (*Parrotomyia*) *modii* Lewis, 1978; *S.* (*Parrotomyia*) *rudnicki* Lewis, 1978; *Sergentomyia* (*Sergentomyia*) *dentata* Sinton, 1933; *S.* (*Sergentomyia*) *pondicherriensis* Srinivasan & Jambulingam, 2010; and *S.* (*Sergentomyia*) *punjabensis* Sinton, 1933.

## Discussion

The present keys were prepared after an extensive morphological screening of specimens collected from different regions of Sri Lanka and reference collection at the Department of Parasitology, Faculty of Medicine of the University of Kelaniya, Sri Lanka. To our knowledge, these are the first keys prepared for Sri Lankan sand fly fauna, and include 20 species of sand flies from two genera (*Phlebotomus* and *Sergentomyia*) reported in the country [19]. The simplified keys allows distinguishing each genus, subgenus and species based on the most important morphological features.

Female and male sand flies differ considerably. Therefore, two keys, representing male and female flies, are presented here for each genus. The sex of the sand fly can be identified using external features. Male sand flies have clasping structures consisting of gonocoxites, gonostyles, parameres, cerci, and aedeagus at the terminal part of the abdomen, but females do not have such clasping structures; the end of the abdomen is blunt, only the cercus is present as an appendage. *Phlebotomus* spp. do not have teeth or a pigment patch [6]. However, in some species of the genus *Phlebotomus,* the cibarium may consist of small scattered spicules [6, 7]. Therefore, it is important to identify the genus based on the combination of all mentioned features. Although the orientation of the hairs and the shape of hair sockets in the posterior margin of abdominal tergites 2 to 6 is mentioned as a diagnostic feature to genus level differentiation [6, 7, 23], our observations suggest that these are not ideal characters to separate the two genera in practical situations as these specimens need a clearing process before mounting; once the specimen is cleared, the hairs may not be found on the abdomen. On the other hand, observation of specimens confirmed to belong to the genus *Sergentomyia* according to wing and cibarium features were found to have hair sockets similar in shape to those in *Phlebotomus* spp. Therefore, this feature is not included in the present key for differentiation of the two genera.

Separation of male sand flies into genera was solely based on the arrangement of spines on the gonostyle. In most of the published keys, the style in species of the genus *Sergentomyia* has been characterized by having only terminal spines. However, *S. arboris*, *S. dentata* and *S. zeylanica*do not comply with this. Therefore, this is a considerable drawback in many of the identification keys often leading to misidentifications. Hence, in the present key, the above limitation has been resolved by mentioning that, if the spines are subterminal they are often found in pairs in *Sergentomyia*. In contrast, spines on the gonostyle of *Phlebotomus* spp. are not arranged in pairs.

The distinct species status of *P. marginatus* is somewhat debatable as the only feature which separates this species from *P. argentipes* is the difference in the color on the dorsal side of the thorax in females [10]. The type-specimen (a female specimen), was not found in the National Insect Collection of Zoological Survey of India (Indian Museum), where it was initially deposited in 1912 [20]. This species is often considered to be a variety of *P. argentipes* [20, 24]. However, this species cannot be considered as an invalid species or a synonym for *P. argentipes* until the type-specimen is found or new specimens are collected from the type-locality according to International Code for Zoological Nomenclature (ICZN) guidelines [22]. A previous study suggests the presence of a morphologically distinct population of *P*. *argentipes* in Sri Lanka [24]. This is likely to be the species denoted as *P. marginatus*. However, more extensive studies are required to confirm the absence of another variant of *P. argentipes* complying with the previous brief description of *P. marginatus*.

One of the main limitations of the keys is that the males are completely unknown for *P. marginatus*, *S. bailyi*, *S. jamesi* and *S. modii* [6]. Therefore, species characterization was not illustrated in the identification key based on male morphology. Furthermore, for a few species, the biological specimens were not available in archives or they were not of good quality to take a clear photograph. The keys were supplemented with illustrations adapted from published literature [6, 7, 21]. Morphometric and meristic analysis during the present study did not reveal any differential features for *S. babu insularis* and *S. baghdadis*. Our study and early studies suggest a close affinity of these two species [25]. However, we cannot consider these two as variations of the same species due to considerable morphological differences observed in females. Therefore, these two species are not separated at the species level in the key based on male morphology. Several species other than *P. argentipes*, the vector of *Leishmania donovani*, and some other arboviruses such as the Chandipura virus in the South Asian region [26, 27], are potential vectors for some disease agents. *Phlebotomus stantoni* is suspected to have some potential in transmitting *Trypanosoma* spp. [28] while *P. salehi* is a known secondary vector for *Leishmania major* in India and Iran [29, 30]. Among the *Sergentomyia* species, *S. punjabensis* and *S. bailyi* are suspected to be vectors for the Chandipura virus [2, 31], while *S. barraudi* is suspected to be a vector for *Leishmania martiniquensis* in Thailand [32]. Therefore, both males and females of all medically important and commonly found sand flies can be identified using the present keys.

There may be minor changes in the morphological characterization of the same species identified from different geographical locations. The morphology of *P. salehi* reported from Sri Lanka [17] is almost similar to that of sand flies reported from Pakistan [7] with some minor changes such as comparatively larger AIII and higher wing index (R2/R 2+3) values. The antennal ascoid (s*ensilla chaetica*) of the AIV was also reported to be comparatively larger [17]. Therefore, in case of doubt, it is essential to consult published literature with detailed species descriptions and/or resolve with molecular-based modern tools.

Recent investigations have emphasized the effectiveness and accuracy of the use of different genetic-based tools such as genetic markers (ITS2, *cytb*-*nad*1) for molecular recognition [33, 34]. In addition, protein-based characterization by matrix-assisted laser desorption/ionization time of flight mass spectrometry (MALDI-TOF MS) for sand flies [35]. However, at field-based uses, molecular or protein-based approaches are not feasible. Therefore, the microscopy examination of morphological characters in basic structures is indispensable and still, the identification of sand flies is achieved through morphology-based features in the pharynx, spermathecae, cibarium of adult females and terminalia in adult males [23].

Identification of sand flies requires a high level of expertise in each of the aspects; dissection, mounting, and accurate recognition of relevant morphological features. Sand flies have been described based on both external and internal morphological characteristics. Even the separation at the generic level involves the observation of internal features such as cibarium. Furthermore, species-level identification requires the observation of features such as the pharynx, spermatheca and genital filaments, which is very difficult due to the presence of hairs and scales on the abdomen of sand flies. Therefore, dehydration with ethanol and xylene, followed by clearing with lactophenol is critical to have a clear view of the internal organs. Hence, all these procedures must be followed accurately for proper identification. If the fixing procedure is completely efficient, important features such as spermatheca may be recognized easily under high-power light microscope. However, unless the specimen is cleared using lactophenol, the specimen should be dissected to reveal the spermatheca. This can be done by placing a fine dissecting needle at the base of the cerci and dragging downward while holding the abdomen with another needle. Almost all features required for the species-level identification are visible under medium (10×) or high magnification (40×) objective of a light microscope. Nevertheless, the observation of cibarium and pharynx, especially for counting teeth may require an oil-immersion objective (100×).

In Sri Lanka, even though the first record of sand flies was documented in 1910 [36], field identification of this medically important group of insects has been carried out referring to the basic features and published literature from other countries thus far. Therefore, this may have contributed as a limiting factor to develop the research interest and capacity among scientists in the country on vector biology, bionomics, vector-parasite interactions and taxonomy-based research as identification is the fundamental requirement for anyone dealing with medically or veterinary important insects. The morphological identification keys presented here are accompanied by digital photographs taken from original specimens and illustrations adapted from published literature [6, 7, 21], which make the keys more user-friendly and facilitating accurate identification of species. The keys are meant as an aid to the rapid identification of sand fly species recorded in Sri Lanka. Hence, this study would strengthen the research capacity and act as a catalyst to focus on the distribution and taxonomy-based studies.

## Conclusions

The keys based on male and female morphology enable the identification of phlebotomine sand flies reported from Sri Lanka since 1910, at the genus and species levels. To our knowledge, this is the first attempt to provide a key to sand flies recorded in the country. The simplified keys, along with photographs taken from specimens and drawings would be beneficial to the health staff, entomologists and research staff who deal with leishmaniasis control and vector-related studies. Hence, the present work would contribute towards improving research capacity among researchers in Sri Lanka as the identification is a fundamental requirement for anyone dealing with medically important insects.

## Supplementary information

**Additional file 1: Table S1.** Details of examined specimens for the preparation of the morphological identification key.

## Data Availability

Data supporting the conclusions of this article are included within the article.

## Abbreviations

ICZN: International Code for Zoological Nomenclature
cytb-nd1: cytochrome *b*-nd1 gene
ITS2: internal transcribed spacer 2 region
