# Supplementary material for: Morphological identification keys for adults of sand flies (Diptera: Psychodidae) in Sri Lanka
Source: Parasit Vectors. 2020 Sep 7;13:450. doi: 10.1186/s13071-020-04305-w (PMC7487486; doi:10.1186/s13071-020-04305-w)
Supplement: Supplementary file 1 — Additional file 1: Table S1. Details of examined specimens for the preparation of the morphological identification key. [file 13071_2020_4305_MOESM1_ESM.docx]

**Additional file 1: Table S1**

Details of specimens examined for the development of the morphological identification key

| **Specimens** | **Collected person and year** | **Locality** | **Acquisition** |
| --- | --- | --- | --- |
| *Phlebotomus argentipes* ♂ & ♀ | Lane, 1987 | Deiyanwela, Kandy, Sri Lanka | Via collaboration |
| *Phlebotomus argentipes sensu stricto* ♂ | Wijerathna and Gunathilaka, 2017 | Kurunegala, Sri Lanka | Collected by authors |
| *Phlebotomus argentipes sensu stricto* ♀ | Wijerathna and Gunathilaka, 2017 | Kurunegala, Sri Lanka | Collected by authors |
| *Phlebotomus argentipes sensu stricto* ♀ | MOH, Medawachchiya, 2019 | Medawachchiya, Anuradhapura, Sri Lanka | Referred to authors for identification |
| *Phlebotomus argentipes sensu stricto ♂ & ♀* | Wijerathna, 2019 | Hambanthota, Sri Lanka | Collected by authors |
| *Phlebotomus argentipes sensu stricto* ♂ & ♀ | AMC, 2019 | Mannar, Sri Lanka | Referred to authors for identification |
| *Phlebotomus argentipes sensu stricto* ♂ & ♀ | AMC, 2019 | Trincomalee, Sri Lanka | Referred to authors for identification |
| *Phlebotomus glaucus ♂ &* ♀ | P.J. Jude, 2019 | Jaffna, Sri Lanka | Referred to authors for identification |
| *Phlebotomus glaucus ♂ &* ♀ | P.J. Jude, 2019 | Mannar, Sri Lanka | Referred to authors for identification |
| *Phlebotomus glaucus* ♂ | Wijerathna, 2019 | Ragama, Sri Lanka | Collected by authors |
| *Phlebotomus stantoni* ♀ | Carter, 1934 | Kalalgoda, Sri Lanka | Via collaboration |
| *Phlebotomus stantoni* ♂^#^ | Unknown, 1857 | India | Via collaboration |
| *Phlebotomus stantoni* ♀*^#^* | Unknown, 1857 | Mysore, India | Via collaboration |
| *Phlebotomus stantoni* ♂ *^#^* | Unknown, 1857 | Mysore, India | Via collaboration |
| *Phlebotomus arboris* ♂  (*Sergentomyia arboris*)* | Carter, 1932 | Katuwawala, Sri Lanka | Via collaboration |
| *Phlebotomus arboris* ♂  (*Sergentomyia arboris*)* | Carter, 1932 | Godigomuwa, Sri Lanka | Via collaboration |
| *Phlebotomus arboris* ♀  (*Sergentomyia arboris*)* | Carter, 1934 | Kalugoda, Sri Lanka | Via collaboration |
| *Phlebotomus babu insularis* ♂  (*Serentomyia babu insularis*) | Carter, 1933 | Depanama, Pannipitiya, Sri Lanka | Via collaboration |
| *Phlebotomus babu insularis* ♀  (*Serentomyia babu insularis*) | Carter, 1933 | Depanama, Pannipitiya, Sri Lanka | Via collaboration |
| *Phlebotomus minutus var. antennatus ♀*  (*Sergentomyia punjabiensis*) | Sinton, 1929 | Unknown | Via collaboration |
| *Phlebotomus minutus var. antennatus ♀*  (*Sergentomyia punjabiensis*) | Unknown, 1831 | Unknown | Via collaboration |
| *Phlebotomus minutus var. antennatus ♀*  (*Sergentomyia punjabiensis*)^#^ | Unknown, 1932 | Patiala, India | Via collaboration |
| *Phlebotomus antennatus* ***♀***  (*Sergentomyia punjabiensis*)*^#^* | Unknown, 1923 | Nagpoor, India | Via collaboration |
| *Sergentomyia punjabiensis ♂* | Wijerathna and Gunathilaka, 2017 | Kurunegala, Sri Lanka | Via collaboration |
| *Sergentomyia punjabiensis ♀* | Wijerathna and Gunathilaka, 2017 | Kurunegala, Sri Lanka | Via collaboration |
| *Phlebotomus zeylanicus* ♂  (*Serentomyia zeylanica*) | Carter, 1932 | Godigomuwa, Sri Lanka | Via collaboration |
| *Phlebotomus zeylanicus* ♀  (*Serentomyia zeylanica*) | Carter, 1932 | Godigomuwa, Sri Lanka | Via collaboration |
| *Phlebotomus zeylanicus* ♀  (*Serentomyia zeylanica*) | Carter, 1932 | Godigomuwa, Sri Lanka | Via collaboration |
| *Phlebotomus zeylanicus* ♂  (*Serentomyia zeylanica*) | Carter, 1934 | Depanama, Pannipitiya, Sri Lanka | Via collaboration |
| *Sergentomyia zeylanica* ♂ | Wijerathna, 2017 | Wandurawa, Sri Lanka | Collected by authors |
| *Sergentomyia zeylanica ♀* | Wijerathna, 2017 | Wandurawa, Sri Lanka | Collected by authors |
| *Sergentomyia zeylanica* ♂ | Gunathilaka, 2018 | Bemmulla, Sri Lanka | Collected by authors |
| *Sergentomyia zeylanica ♀* | Gunathilaka, 2018 | Bemmulla, Sri Lanka | Collected by authors |
| *Sergentomyia zeylanica* ♂ | MOH, Mirigama, 2019 | Kal-eliya, Sri Lanka | Referred to authors for identification |
| *Sergentomyia zeylanica*♀ | MOH, Mirigama, 2019 | Kal-eliya, Sri Lanka | Referred to authors for identification |
| *Sergentomyia zeylanica* ♂ | Mallawarachchi, 2019 | Kahatapitiya, Sri Lanka | Referred to authors for identification |
| *Sergentomyia zeylanica*♀ | Mallawarachchi, 2019 | Kahatapitiya, Sri Lanka | Referred to authors for identification |
| *Sergentomyia zeylanica*♂ | Regional Entomologist, Gampaha, 2019 | Gampaha, Sri Lanka | Referred to authors for identification |

^#^Specimens collected outside of Sri Lanka available at the archives of the Department of Parasitology used in the species confirmation. AMC: Antimalarial campaign; MOH: Medical Officer of Health; Regional Entomologist
